# Editorial: Gut microbiota and chemotherapy resistance of colorectal cancer

**DOI:** 10.3389/fgstr.2023.1167322

**Published:** 2023-03-13

**Authors:** Yijia Wang, Xuehua Wan, Shaobin Hou

**Affiliations:** ^1^ Laboratory of Oncologic Molecular Medicine, Tianjin Union Medical Center, Nankai University, Tianjin, China; ^2^ TEDA Institute of Biological Sciences and Biotechnology, Nankai University, Tianjin, China; ^3^ Advanced Studies in Genomics, Proteomics, and Bioinformatics, University of Hawaii At Manoa, Honolulu, HI, United States

**Keywords:** gut microbiota, chemotherapy resistance, colorectal cancer, tumor microenvironment, metabolite

Gut microbiota play a crucial role in the development and progression of colorectal cancer (CRC). Microbes or their metabolites interact with the intestinal mucosal surfaces directly, which regulate the host immune response and some signaling pathways of the intestinal epithelial cells. These regulation effects change the immune escape ability of tumor cells and some key proteins in the signaling pathways that are related to chemotherapy resistance.

Chen et al. found that a higher gut microbiota diversity was associated with more favorable treatment outcomes in patients with metastatic CRC. The authors enrolled 110 patients with metastatic CRC, who were treated with cetuximab or bevacizumab. *Klebsiella quasipneumoniae* exhibited greater fold change in abundance in the progressive disease (PD) group than in the partial response (PR) group. The *Lactobacillus* and *Bifidobacterium* species exhibited higher abundance in the PD group. The abundance of *Fusobacterium nucleatum* was approximately 32 times higher in the PD group than in the PR group. These bacteria may be potential markers to predict the curative effects of cetuximab or bevacizumab. A prospective, randomized study with a larger sample size and greater number of stool samples is necessary to validate the correlation between microbiota and targeted therapy in mCRC.

Sun et al. distinguished the differences of gut microbiota between CRC with and without metastases. The results showed that the microbial composition (diversity) of the healthy control group was better than the CRC patients group, while the same difference was also found between the tumor and metastasis groups. Some changes of bacterial abundances were also found to be characteristic of metastatic CRC. Therefore, the analysis of gut microbiota can serve as a supplementary biological basis for the diagnosis and treatment of metastatic colorectal cancer, which may offer the potential to develop non-invasive diagnostic tests.

In their opinion article, Jia et al. discussed the close relationship between the enteric microbiota, enteric nervous system, and CRC tumorigenesis, which may provide a new perspective for CRC treatment.

Qin et al. established a radioresistant colorectal cancer cell line developed from the parental HCT116 cell. Transcriptomics analyses were performed to search for the underlying genes that contribute to radioresistance and to investigate its association with the prognosis of CRC patients. The authors built a risk score model with five radioresistance genes, including TNFRSF13C, CD36, ANGPTL4, LAMB3, and SERPINA1, to predict prognosis after radiotherapy for CRC.

Zhu et al. assessed the microbial composition and diversity of sporadic CRC tumors with varying MutL protein homolog 1 (MLH1) status and the effects of functional genes related to bacterial markers and clinical diagnostic prediction. The authors constructed a high-accuracy model to detect and evaluate the area under the receiver operating characteristic curve with candidate biomarkers. The study included 23 patients with negative/defective MLH1 (DM group) and 22 patients with positive/intact MLH1. The authors found that the genera *Lachnoclostridium* and *Coprococcus* as key species may be crucial biomarkers for the non-invasive diagnostic prediction of DM in patients with sporadic CRC in the future.

In general, the authors of these articles found some characteristic bacteria in various types of CRC or chemotherapy prognosis. These bacteria and their diversity were used to created potential prediction markers in CRC identification or chemotherapy selection. Considering the critical role of gut microbiota in the tumor microenvironment, these findings help us to further understand the role of gut microbiota in CRC chemotherapy. However, the investigation of the mechanisms of how gut microbiota affect the resistance of chemotherapy is still lacking in these studies. Many studies focus on the level of strain but ignore the metabolite or antigen of bacteria. Future investigations may focus on the interaction between the metabolite of bacteria and the intestines or tumors to explore the role of gut microbiota on chemotherapy.

## Author contributions

All authors listed have made a substantial, direct, and intellectual contribution to the work and approved it for publication.

